# Automated Workflow for Processing and Classifying Dental Radiographs: A Hands-On Approach

**DOI:** 10.7759/cureus.84816

**Published:** 2025-05-26

**Authors:** Rajmohan Sivamani Chidambaram, Ragavesh Dhandapani, Sudha Rajmohan

**Affiliations:** 1 Department of Prosthodontics, Oman Dental College, Muscat, OMN; 2 Department of Electrical and Communication Engineering, National University of Science and Technology, Muscat, OMN; 3 Department of Conservative Dentistry, Oman Dental College, Muscat, OMN

**Keywords:** computer-assisted, convolutional neural networks, machine learning, radiographic image interpretation, radiography dental

## Abstract

Background

Classifying large dental radiographic datasets enables efficient data management and retrieval, facilitating quick access to specific types of radiographs for clinical or research purposes. It also supports advanced analytics, research, and the development of Artificial Intelligence (AI) tools. This study aimed to develop an automated workflow to improve the efficiency of dental radiograph classification. The workflow covers the entire process, from retrieving Digital Imaging and Communication in Medicine (DICOM) files to converting them into Joint Photographic Experts Group (JPEG) format and classifying them using Convolutional Neural Networks (CNNs) on a large dataset.

Materials and methods

This cross-sectional machine learning study was conducted using 48,329 dental radiographs to develop an automated classification workflow using CNNs. The workflow involved retrieving 48,329 DICOM files, standardizing them to a uniform size, and converting them to JPEG using the Pydicom library. Image preprocessing, including normalization, prepared the images for machine learning analysis. Various models, such as ResNet-50, AlexNet, and custom CNN models, were trained, validated, and tested on distinct datasets.

Results

These models were then deployed to classify a dataset of 48329 images. AlexNet demonstrated the highest performance, with a 95.98% detection rate and no errors, while ResNet-50 achieved 92.3% accuracy with 194 errors, and the custom CNN model showed a 77.25% detection rate with 1,623 errors.

Conclusion

The study established an effective automated workflow for dental radiograph classification, demonstrating that CNN models significantly improve classification accuracy and efficiency.

## Introduction

Digital Imaging and Communication in Medicine (DICOM) has revolutionized radiography by standardizing medical imaging. Dental radiography presents specific challenges like large file sizes and complex metadata, which impact efficiency. Manual classification is often error-prone, prompting the use of automation solutions. Convolutional Neural Networks (CNNs) offer an effective tool for image classification, with models like AlexNet and ResNet excelling in medical imaging tasks. This paper proposes a workflow from DICOM retrieval to classification using CNN models.

The management of dental radiographs for research encounters several operational challenges, primarily stemming from the manual handling of DICOM files, which includes their identification, extraction, and organization [1-3]. This process is often inefficient and error-prone. Additionally, the lack of standardized filenames further complicates the efficient search and retrieval of specific radiographs, slowing down the process significantly. Manual classification of these radiographs introduces subjectivity, adversely impacting both diagnostic accuracy and the utility of the research [4]. To address these issues, various automation solutions have been developed. Researchers have explored algorithms for identifying and extracting relevant metadata from DICOM files, facilitating automated organization and tagging. Proposals for standardized naming schemes based on this metadata aim to improve the retrieval efficiency of these radiographs [5,6].

The application of CNNs in image recognition has demonstrated exceptional performance enhancements across various domains [7], including medical imaging [8]. CNNs, characterized by their ability to automatically detect important features without the need for manual extraction, are particularly suited for processing complex image data such as that found in dental radiographs. However, the transition from DICOM images, which contain rich metadata and are encoded in a format suitable for medical use, to a more universally usable format like JPEG involves critical preprocessing steps [9,10]. These steps include retrieval, standardization of image dimensions and intensity scales, and conversion, each of which must preserve the diagnostic quality of the images. Different radiograph types, such as Bitewing, orthopantomogram (OPG), cephalometric radiographs (Ceph), and posterior intraoral periapical (Postiopa), present unique challenges due to variations in anatomical focus, image scale, and orientation. Accurately classifying these requires the model to distinguish subtle differences in anatomical structures and image features. The effective classification of these images into their respective categories not only enhances the diagnostic workflow but also aids in automating and streamlining the storage and retrieval processes in dental practice management systems [11-13].

This study aims to develop an automated workflow for processing and classifying dental radiographs, beginning with retrieving and standardizing DICOM images, then converting them to JPEG format, and culminating in the classification using a CNN. The models used (including ResNet-50 [13], AlexNet [14], and the custom model) were trained, validated, and tested on distinct dataset partitions. The primary objectives are to evaluate the efficiency and accuracy of this workflow in maintaining the diagnostic quality of images throughout the processing pipeline and to assess the performance of the CNN in accurately classifying various types of dental radiographs. The remainder of the paper is organized as follows: Section 2 describes the data acquisition process, DICOM standardization, JPEG conversion, and CNN model development, including dataset preparation, training, testing, and validation. Section 3 presents the performance of custom CNN, ResNet-50, and AlexNet models. Section 4 discusses the findings, comparing models with existing methods and suggesting improvements for challenging radiograph types. Section 5 concludes the study, highlighting the model’s performance and future work directions.

## Materials and methods

**Figure 1:**
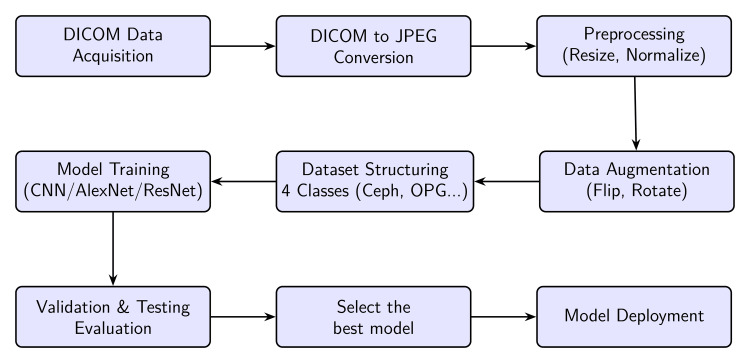
Overview of the data processing workflow

The block diagram in Figure 1 shows an overview of the work, highlighting the key stages from image acquisition to model deployment. Each block represents a crucial step in the workflow. The following sections provide a detailed explanation of each component.

Data acquisition and image processing

Dental X-rays were captured daily by the attending dentist during routine clinical procedures and stored on the X-ray machine’s server. We commenced with setting up a system to scan and retrieve DICOM files from medical imaging devices while ensuring compliance with secure access protocols to protect data privacy. The DICOM files were organized based on relevant metadata, such as acquisition date and study type, facilitating easy access and structured analysis in subsequent stages. Initial quality checks were performed to ensure the integrity of the DICOM files, including verifying completeness and absence of corruption and confirming the presence of critical metadata like ’Study Date’ and ’Bits Stored’.

These files were then standardized to uniform orientation and size through necessary adjustments such as rotation, cropping, or resizing. Following standardization, the DICOM images were converted to the JPEG format using the Pydicom library, with particular attention to preserving information, especially where the original images had a higher bit depth than typical JPEG files. During this conversion, a coding mechanism adjusted the ’Bits Stored’ to maintain image data integrity.

Dataset preparation

To facilitate effective model training, the JPEG radiographic images underwent a standardized preprocessing pipeline. This included resizing all images to 224×224 pixels to comply with the input dimensions required by pre-trained convolutional neural networks such as ResNet-50 and AlexNet. Pixel intensity values were normalized using ImageNet statistics (mean = (0.485, 0.456, 0.406), std = (0.229, 0.224, 0.225)) to align with the pretrained model expectations. Furthermore, data augmentation techniques, including random horizontal flipping, rotation (±15°), random resized cropping (scale = 0.8-1.0), and color jittering (brightness and contrast adjustments up to ±20%), were applied to the training set. These steps were designed to enhance the model’s robustness, prevent overfitting, and improve generalizability across diverse radiographic types.

**Figure 2:**
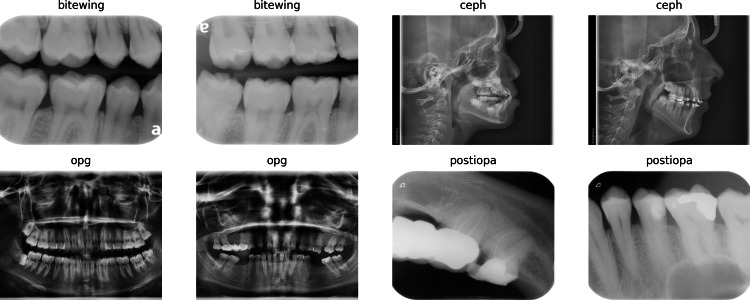
Samples of radiographs used for training, validation, and testing

The dataset used for developing this model was a custom collection of dental X-ray images, which were categorized into four classes: Bitewing, Ceph, OPG, and Postiopa. A few samples of radiographs used in this work are shown in Figure 2. The images were loaded from a directory using ‘ImageFolder‘ from ‘torchvision.datasets‘ and were transformed into tensors using ‘transforms.Compose‘, which normalized the pixel values to a range of -1 to 1. During the data preparation phase, the images were resized to a fixed size of 255x255 pixels using OpenCV. The training set was constructed by looping through the directory containing the images and appending the image data and its corresponding class label to a list. This training dataset was converted into a DataLoader for efficient batch processing during model training.

CNN preparation and execution

A suitable CNN architecture was selected based on the specific needs of the study, which could involve adapting pre-existing models like ResNet-50 [13] or AlexNet [14], or developing a custom model. The dataset was split into training, testing, and validation sets. We used a 70:30 split for training and validation/testing, respectively. The CNN was trained using the training set, with continuous evaluation on the validation set to fine-tune hyperparameters and prevent overfitting. The hyperparameters used were 15 epochs, a batch size of 32, a learning rate of 0.001, and a Stochastic Gradient Descent (SGD) optimizer with 0.9 momentum. The entire network was fine-tuned, with all layers unfrozen during training.

Custom model

**Figure 3:**
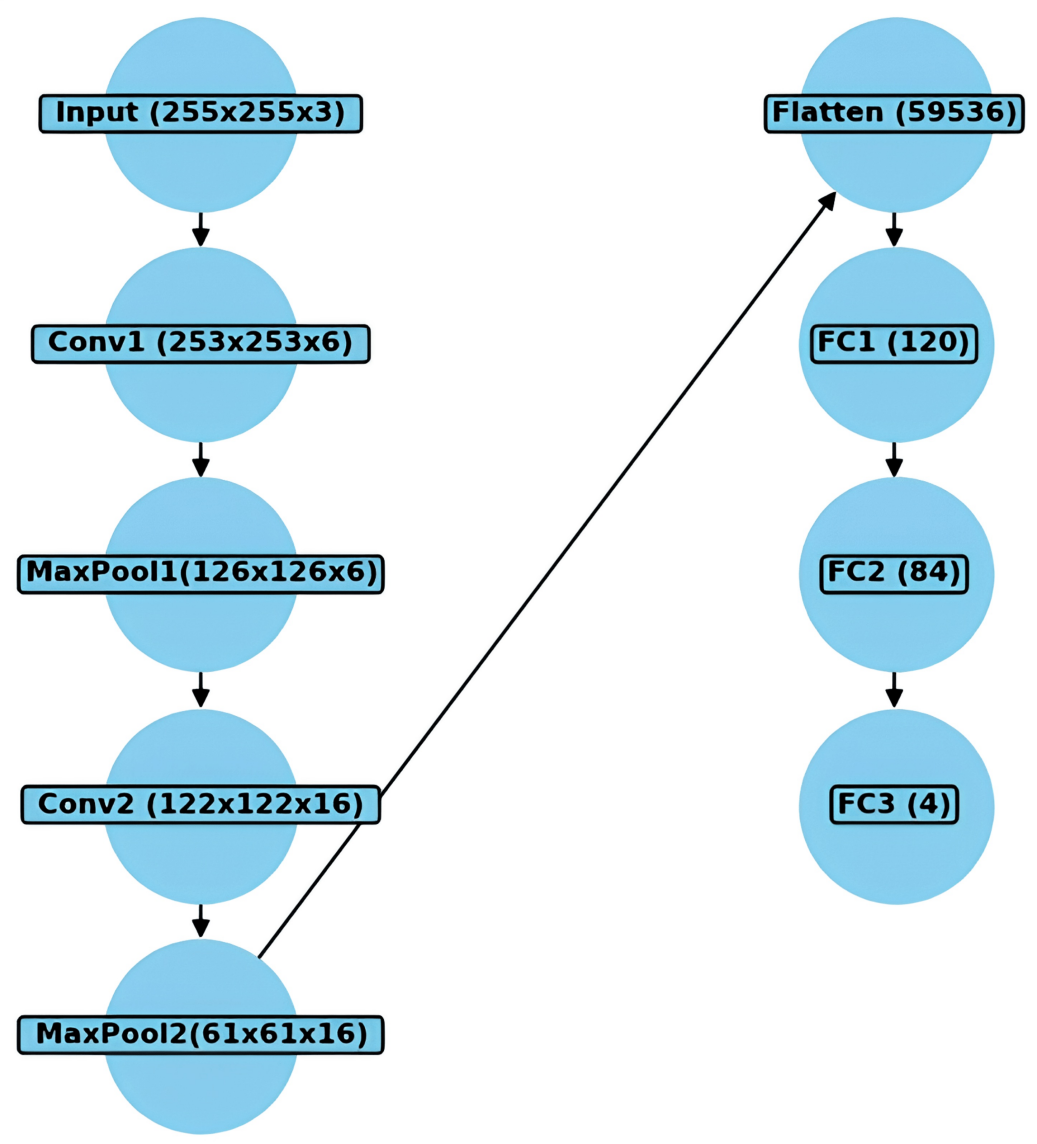
Model architecture of the custom CNN model CNN: Convolutional Neural Network

Figure 3 shows that the CNN model was developed using the PyTorch framework to classify dental X-ray images into four categories: Bitewing, Ceph, OPG, and Postiopa. Several essential libraries were employed to implement the model. PyTorch, as the core framework, provided automatic differentiation, graphics processing unit (GPU) support, and functionalities for neural network operations such as convolutional layers and loss functions. ‘torchvision‘ was used for transforming images into tensors and normalizing them, while OpenCV (‘cv2‘) handled image loading and preprocessing, resizing the images to a uniform size of 255x255 pixels. NumPy assisted in array operations and image normalization, while Matplotlib enabled visualization of the images at various preprocessing stages. Finally, ‘tqdm‘ provided progress tracking when loading the dataset, offering visual feedback during the data preparation phase.

The CNN model consists of two convolutional layers, max-pooling, and three fully connected layers. The first convolutional layer had 3 input channels and 6 output channels with a kernel size of 3, while the second convolutional layer had 6 input channels and 16 output channels with a kernel size of 5. The ReLU activation function was used after each convolutional layer to introduce non-linearity. The max-pooling layers reduced the dimensionality of the feature maps, which were then flattened into a 1D tensor and passed through three fully connected layers. The final layer outputted four values, representing the probabilities for each image class. ReLU was applied in the hidden layers, and the network was optimized using backpropagation through the ‘SGD‘ optimizer.

The model was trained for 100 epochs, ensuring sufficient network exposure to the dataset. A batch size of 4 was selected, meaning the model processed four images at a time before updating its weights. The learning rate was set to 0.0005 to ensure a gradual optimization of the model’s parameters. The Stochastic Gradient Descent (SGD) optimizer was used to minimize the loss function, ‘CrossEntropyLoss‘, which calculates the difference between the predicted and actual class probabilities. These hyperparameters ensured that the model learned efficiently from the dataset without overfitting.

Model validation and clinical integration

The model was rigorously tested on an independent set of images not used during the training phase. Dental professionals provided clinical insights by reviewing the model’s predictions and validating its accuracy and applicability in real-world settings. Subsequently, a user interface was developed, and the model was integrated into the clinical workflow.

Performance metrics and time taken

The entire process of retrieving, processing, and converting 48329 DICOM files into JPEG format was accomplished in six hours. All models were developed and evaluated using the same dataset, standardized into training, testing, and validation subsets to ensure consistent and reliable model performance.

Monitoring, maintenance, and expected outcomes

The model was continuously monitored to detect any performance issues or data drift over time. A protocol for periodic retraining or fine-tuning of the model with new data was established to maintain its relevance and accuracy. The expected outcomes of this methodology included the development of a robust automated workflow for processing dental radiographs, creating a high-quality image dataset, an accurate classification model, and enhanced clinical and educational tools that are scalable and adaptable.

Consent and compliance protocol

The study was conducted in accordance with the research guidelines and approved by the Ethics Committee of Oman Dental College. The study’s compliance and oversight procedures aligned with relevant laws and were continuously monitored by the Institutional Review Board (IRB) to ensure ethical standards and regulatory requirements were met, thus maintaining the study’s integrity and data protection. In this investigation, informed consent was not required due to the absence of direct patient interaction, and strict adherence to data privacy principles was maintained to ensure all patient identifiers were excluded from the radiographic data. The confidentiality and anonymity of data were rigorously upheld throughout the research process. Access to personal data was limited to essential research team members, who were all trained in handling sensitive information. All radiographic data were de-identified early in the process, with identifiable markers removed or obscured, and results were aggregated to prevent disclosure of individual information. Data security was robust, with all de-identified radiographs and analysis results stored on secure, password-protected servers. The data was retained according to institutional and ethical guidelines and then securely destroyed. Secure, encrypted methods were used for data transfer and sharing within the team and with external collaborators, adhering to stringent data-sharing agreements that maintained the original study’s confidentiality levels.

Data analysis

Statistical analysis was conducted using chi-square tests for independence to assess differences in classification accuracy between the CNN models (ResNet-50 and AlexNet). Chi-square tests evaluated the dependency between model accuracy and radiograph types by comparing observed and expected frequencies.

## Results

Table 1 summarizes the overall classification accuracy of all models evaluated in our study. The breakdown of the classification percentage is shown in Table 2. AlexNet achieved perfect classification with a 95.98% and no errors. ResNet-50 followed with a high accuracy of 92.71% and only 194 misclassifications, while the custom CNN model had a lower classification rate of 78.34% with 525 misclassifications.

**Table 1:** Summary of image classification by method Data are represented as the number of correctly classified images (N) and classification accuracy as a percentage (%). Statistical analysis was performed using the chi-square test to compare model performance across radiograph types. The total chi-square statistic was 159.8 with a p-value < 0.0001, indicating a highly statistically significant difference. A p-value < 0.05 was considered statistically significant.

Classification models	Total Images	Correct Classification	Classification Accuracy
Custom CNN	48329	37865	78.34%
ResNet-50	48329	44807	92.71%
AlexNet	48329	46387	95.98%

Table 2 provides a detailed breakdown of CNN’s performance by radiograph type. CNN performed best with OPG radiographs, achieving a 98.44% classification rate. It was also notably effective in Ceph radiographs, albeit with a classification rate exceeding 100% (102.24%), likely due to over-classification. In contrast, CNN performed below overall accuracy with Postiopa radiographs and Other Types, with classification rates of 74.07% and 0%, respectively, the latter indicating a complete miss in classifying any images of that type. Bitewing radiographs had a relatively high classification rate of 88.32%. The custom CNN model classified 34 images of other types of radiographs as Ceph, and 491 images were misclassified as Postiopa. The ResNet-50 model misclassified 194 images of other types of images, as Postiopa and Alexnet have no error in classification.

**Table 2:** Breakdown of classification percentage by radiograph type for all classifiers Data are represented as the number of correctly classified images (N) and classification accuracy as a percentage (%) for each model. The total chi-square statistic was 159.8 with a p-value of < 0.0001, indicating a highly statistically significant difference. A p-value of < 0.05 was considered statistically significant.

Radiograph	Total	Custom CNN Model			ResNet-50 Model			AlexNet Model		
		Classified	Error	%	Classified	Error	%	Classified	Error	%
Bitewing	9483	8375	0	88.32%	8897	0	93.82%	9450	0	99.65%
OPG	2887	2842	0	98.44%	2885	0	99.93%	2885	0	99.93%
Ceph	1516	1550	34	102.24%	1516	0	100.00%	1503	0	99.14%
Postiopa	33884	25098	491	74.07%	31509	194	92.99%	32549	0	96.06%
Other Types	559	--	--	--	--	--	--	--	--	--
Total	48329	37865	525		44807	194		46387	0	

The accuracy, precision, and recall of each classifier based on radiograph type are shown in Table 3.

**Table 3:** Overall performance comparison of different models CNN: Convolutional Neural Networks; OPG: Orthopantomogram

Classifier	Radiograph	Accuracy	Precision	Recall
Custom CNN	Bitewing	97.71%	100%	88.32%
	OPG	99.91%	100%	98.44%
	Ceph	99.93%	97.81%	100%
	Postiopa	79.80%	98.03%	72.64%
ResNet-50	Bitewing	98.79%	100%	93.82%
	OPG	99.99%	100%	99.93%
	Ceph	100%	100%	100%
	Postiopa	94.30%	99.38%	92.39%
AlexNet	Bitewing	99.91%	100%	99.65%
	OPG	99.99%	100%	99.93%
	Ceph	99.97%	100%	99.14%
	Postiopa	97.24%	100%	96.06%

The performance metrics for the Bitewing radiograph classification using the CNN Model are calculated as follows:

\(\begin{align*} \text{Accuracy} &= \frac{\text{TP} + \text{TN}}{\text{Total Images}} \\ &= \frac{8375 + 38846}{48329} \\ &\approx 97.71\% \end{align*}\)

\( \begin{align*} \text{Precision} &= \frac{\text{TP}}{\text{TP} + \text{FP}} \\ &= \frac{8375}{8375 + 0} \\ &= 100\% \end{align*}\)

\( \begin{align*} \text{Recall} &= \frac{\text{TP}}{\text{TP} + \text{FN}} \\ &= \frac{8375}{8375 + 1108} \\ &\approx 88.32\% \end{align*}\)

where TP is true positive, TN is true negative, FP is false positive, and FN is false negative.

**Figure 4:**
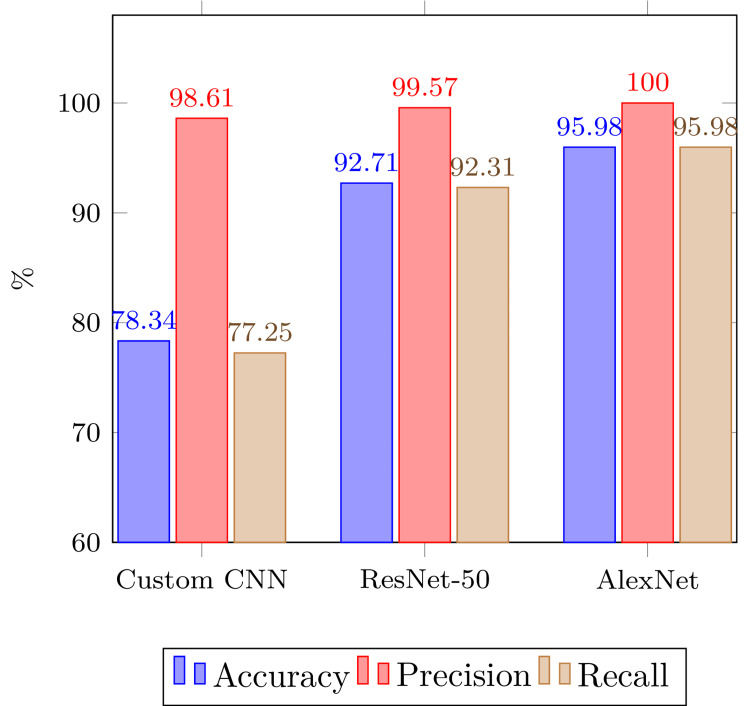
Overall performance comparison of different models

**Figure 5:**
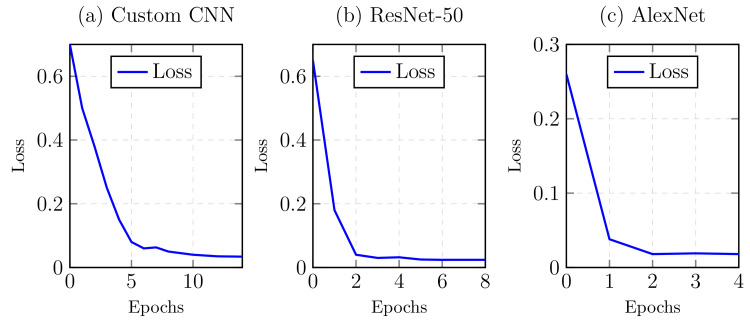
Graphical depiction of training loss for (a) CNN, (b) ResNet-50, and (c) AlexNet models over epochs.

Figure 4 compares performance metrics (accuracy, precision, and F1 Score) between the assessed models, whereas Figure 5 graphically represents the training loss between CNN, Resnet-50, and Alexnet, respectively. All three models demonstrated high effectiveness, with AlexNet leading slightly in accuracy and precision (95.98% and 100%, respectively), followed closely by ResNet-50 and CNN. All models had similar F1 scores, indicating a balance between precision and recall across the models.

**Figure 6:**
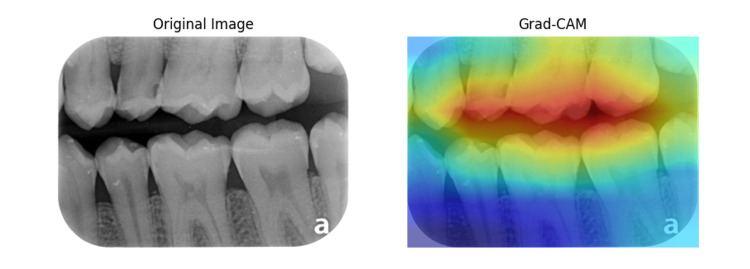
Grad-CAM output images of Bitewing

**Figure 7:**
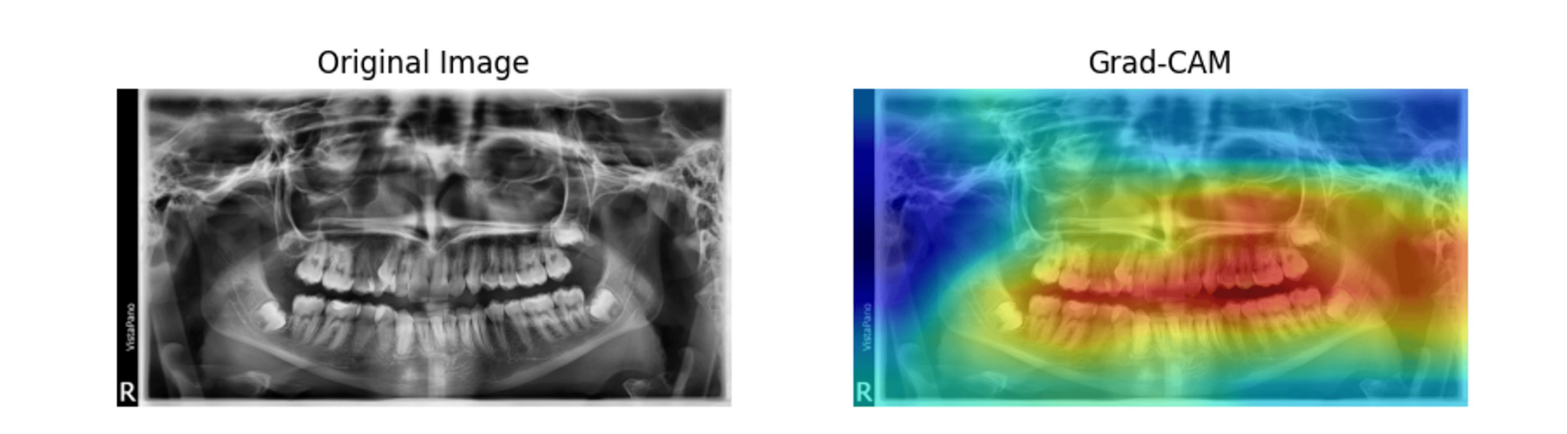
Grad-CAM output images of OPG

Figures 6, 7 show the original dental X-ray image on the left and the Grad-CAM heatmap on the right. The Grad-CAM heatmap highlights the regions of the image that are most important for the classification decision. Table 4 presents the results of a chi-square test for independence, assessing the discrepancy in the number of correct classifications between the CNN and ResNet-50 models across various types of radiographs. The observed values showed that CNN had fewer correct selections than ResNet-50 in all categories except for Ceph radiographs, where CNN slightly outperformed ResNet-50. The largest differences were observed in the Bitewing and Postiopa categories, contributing the most to the chi-square statistic due to higher individual chi-square values (O-E)²/E for both models. The total chi-square statistic of 159.8 with a p-value less than 0.0001 indicates a highly statistically significant difference in classification performance between the two models across all radiograph types.

**Table 4:** Chi-square test for Independence (CNN vs. ResNet-50 classification) accuracy, precision, and recall of different classifiers and radiograph types CNN: Convolutional Neural Networks; OPG: Orthopantomogram

Type of Radiograph	Observed		Expected		Chi-Square	
	CNN	ResNet-50	CNN	ResNet-50	CNN	ResNet-50
Bitewing	8375	8897	7908	9363	27.06	22.87
OPG	2842	2885	2624	3102	18.21	15.40
Ceph	1550	1516	1404	1661	14.99	12.67
Postiopa	25098	31509	25927	30679	26.33	22.27
Total	37865	44807			86.59	73.21

## Discussion

The demonstrated superiority of AlexNet in handling a diverse set of radiographic images without errors in our investigation suggests that it could serve as a reliable tool in clinical settings, reducing the workload of radiologists by accurately classifying images without the need for manual correction. The insights into CNN’s performance can guide further algorithmic adjustments and training to enhance accuracy, particularly for challenging radiograph types like Postiopa and other types. Additionally, the high performance of ResNet-50 indicates its potential as a versatile tool in medical image analysis, though with slight modifications to reduce error rates further.

Comparing our findings with those of Stanley et al., there are both similarities and differences [1]. They explored modality-based discrimination using image-based features and achieved high discrimination results, up to 95.57%, using basis function features. Both studies highlight the effectiveness of advanced image classification techniques. However, while Stanley et al. focused on differentiating between modalities in a relatively small dataset of manually annotated images, our study dealt with a significantly larger dataset [1]. It aimed to classify specific types of radiographs. The use of different feature extraction techniques (basis function features versus deep learning models) and the objectives (modality discrimination versus specific image classification) represent the main differences.

The findings in Cejudo et al.'s study closely align with our work, particularly in terms of the application domain of radiographic classification and the use of deep learning architectures [2]. Cejudo et al. reported high accuracy (>98%) across all models, with ResNet-50 showing the highest performance, which is consistent with our observation of ResNet’s high accuracy (92.71%). However, they used different architectures, such as CapsNet, and employed methods like active learning, which were not explored in our study. The comparison highlights the robustness of ResNet-50 across different studies and datasets and underscores the potential of using varied methodologies like active learning to enhance model training and performance [2].

Krois et al. examined the impact of image context on accuracy in tooth classification on panoramic radiographs [3]. Their approach to manipulating context to improve classification accuracy mirrors our analysis of different radiograph types and their impact on CNN’s performance. Both studies indicate that contextual information significantly affects classification performance. However, Krois et al. focused on how expanding the image context improves classification, a specific aspect that was not directly addressed in our study [3].

Several algorithms have been applied in dental imaging to achieve accurate teeth classification. Notable methods include Fourier descriptors, texture analysis, and Bayesian classifiers [3,4]. These techniques have been instrumental in enhancing the precision of tooth identification processes. In the context of multi-slice CT (MSCT) images, Hosntalab et al. developed a multistage classification algorithm [5]. This method encompasses segmentation, feature extraction, and the classification of teeth using a supervised learning approach. For bitewing radiographs, which are pivotal for tooth identification, artificial intelligence techniques have been extensively applied [5,8]. For instance, Chen et al. utilized a Faster R-CNN framework to identify and label teeth in periapical images from a dataset comprising 1250 images, achieving a high detection accuracy but a lower accuracy in tooth numbering [6].

Tuzoff et al. applied a Faster R-CNN to 1574 panoramic radiographs for tooth detection and numbering, achieving extremely high accuracy and specificity [15]. On a smaller scale, Yuniarti et al. evaluated both bitewing and panoramic images, achieving reasonable accuracy in tooth detection and numbering using a set of 16 images [16]. Estai et al. and Bilgir et al. both utilized CNNs to classify and number teeth in OPGs and panoramic radiographs, respectively, with both studies reporting high precision rates [17,18]. Orhan et al. focused on utilizing cone-beam computed tomography (CBCT) images to detect periapical pathosis, illustrating the broad applicability of CNNs in various diagnostic tasks within dental radiography [19].

The study encountered the following limitations regarding data acquisition and image processing, which could potentially influence the findings. Initially, while DICOM files were organized and standardized, the conversion of these files to JPEG format using the Pydicom library involved adjustments in ’Bits Stored’ to preserve image data integrity. This conversion could introduce subtle variations in image quality not present in the original DICOM format, thus affecting the subsequent analysis. Additionally, in the preparation and execution of CNNs, despite rigorous preprocessing and data augmentation aimed at enhancing model robustness, the inherent variability in image quality and the possibility of overfitting, despite measures to prevent it, could have influenced the performance metrics. Additionally, the selection and adaptation of CNN architectures, whether pre-existing models like ResNet-50 or AlexNet or a custom-built model, might have brought biases based on their respective strengths and weaknesses in handling specific image data types.

## Conclusions

Our findings showed that AlexNet outperformed the other models, achieving a perfect classification precision of 100% with no observable errors. ResNet-50 also demonstrated strong performance, with only minor inaccuracies. In comparison, the conventional CNN model exhibited high variability in performance, particularly with PostiOPA radiographs, where classification accuracy was notably lower, and it failed to identify certain other types. Significant differences in the accuracy of images classified between CNN and ResNet-50 were also revealed. CNN performed suboptimally in most categories, except in Ceph radiographs, where it showed a slight advantage. These findings highlight the superior capabilities of advanced models like AlexNet in radiographic image analysis and underscore the necessity for continuous improvement of CNN algorithms to achieve uniform accuracy across different types of radiographs.
